# Blame it on the weather? The association between pain in fibromyalgia, relative humidity, temperature and barometric pressure

**DOI:** 10.1371/journal.pone.0216902

**Published:** 2019-05-10

**Authors:** Asbjørn J. Fagerlund, Maria Iversen, Andrea Ekeland, Connie Malèn Moen, Per M. Aslaksen

**Affiliations:** 1 Norwegian Center for E-Health Research, Tromsø, Norway; 2 Department of Psychology, UiT The Arctic University of Norway UiT, Tromsø, Norway; 3 Department of Child and Adolescent Psychiatry, The Regional Unit for Eating Disorders, The University Hospital of North Norway, Tromsø, Norway; Parc Sanitari Sant Joan de Déu, SPAIN

## Abstract

Self-reported pain levels in patients with fibromyalgia may change according to weather conditions. Previous studies suggest that low barometric pressure (BMP) is significantly related to increased pain, but that the contribution of changes in BMP has limited clinical relevance. The present study examined whether BMP influenced variability in perceived stress, and if stress levels moderated or mediated the relationship between BMP and pain. Forty-eight patients with fibromyalgia enrolled in a randomized controlled trail (RCT) reported pain and emotional state three times daily with mobile phone messages for a 30-consecutive day period prior to the start of the treatment in the RCT. The patients were unaware that weather data were collected simultaneously with pain and emotional reports. The results showed that lower BMP and increased humidity were significantly associated with increased pain intensity and pain unpleasantness, but only BMP was associated with stress levels. Stress levels moderated the impact of lower BMP on pain intensity significantly, where higher stress was associated with higher pain. Significant individual differences were present shown by a sub-group of patients (n = 8) who reacted opposite compared to the majority of patients (n = 40) with increased pain reports to an increase in BMP. In sum, lower BMP was associated with increased pain and stress levels in the majority of the patients, and stress moderated the relationship between BMP and pain at the group-level. Significant individual differences in response to changes in BMP were present, and the relation between weather and pain may be of clinical relevance at the individual level.

## Introduction

It is a commonly held belief that there is a relationship between weather conditions and pain. In fibromyalgia and other chronic pain conditions this belief is supported by recent data showing that local weather is associated with rates of online searches for pain symptoms [1]. However, previous scientific investigations of the effect of weather on pain have yielded mixed findings. Some studies report that there is a significant association between pain levels, temperature, barometric pressure (BMP) and/or relative air humidity in chronic pain conditions [2–6], whereas other studies failed to find such relationships [7, 8]. Even if significant associations between pain and weather are shown, it is suggested that the clinical relevance of changes in weather conditions on pain in fibromyalgia is negligible at group-levels [9]. Nonetheless, several studies find that lower BMP is significantly related to augmented pain reports in patients with fibromyalgia [3, 5, 9], even if the effect sizes are small. Thus, BMP seems to be the most reliable weather variable for predicting change in pain levels in fibromyalgia. The assumption that BMP is associated with pain is to some extent supported by animal studies, where artificially lowered barometric pressure within natural levels induce increased pain behavior in rats with experimentally induced inflammation or neuropathy [10, 11].

A possibility for explaining the association between BMP and pain is that the weather may modulate affective states that could either mediate or moderate pain levels. It has previously been shown that changes in weather conditions are correlated with self-reported emotional states [12], and that individual differences might be important for the pain and weather relationship even if the direction of this relationship is unclear [4]. Furthermore, self-perceived weather sensitivity is associated with increased pain sensitivity [13]. However, psychological traits assumed to be stable in an individual over time have shown no predictive value for the relation between weather and pain reports [9]. Several studies show that negative stress increase pain reports [14–16], both in experimental studies on healthy volunteers and in chronic pain patients. Thus, we hypothesized that decreased BMP was significantly associated with increased stress reports, and that self-reported stress measured concomitantly with pain should be either a significant moderator or mediator for the relation between BMP and variability in pain reports. Hence, in an exploratory manner, we tested both the possibility that stress could act as a moderator or a mediator for the relationship between BMP and pain levels in patients with fibromyalgia.

## Materials and methods

The present study included data from the 48 (45 female) patients that participated in a previously published RCT that tested the effect of transcranial direct current stimulation (tDCS) on pain in fibromyalgia [17]. The mean age of the sample was 48.6 years (SD = 9.6). The required sample size was calculated for the purpose of the RCT. The patients had to be ≥18 years of age, diagnosed with fibromyalgia (ICD-10 M79.7) according to the ACR-90 criteria [18], and a manual examination of the patients’ tender points was performed before inclusion. All patients who otherwise adhered to the criteria for participation had a positive fibromyalgia diagnostic status. If patients were using prescribed medication, the use had to be stable for 3 months before inclusion. The exclusion criteria included severe psychiatric conditions defined as bipolar disorder, severe depression, and schizophrenia. Additional exclusion criteria consisted of neurological conditions, developmental disorders, pregnancy, and drug abuse. The study was approved by the Regional Committee for Medical and Health Research Ethics (2010/2256) and registered in clinicaltrials.gov (NCT01598181). All patients provided written informed consent by mail. See Fagerlund et al. [17] for additional details.

### Procedure

In our previous study using this sample, the included patients reported pain and emotional measures during a 30-day period before the tDCS treatment began. Thus, the data used in this article is based on the pre-treatment period and does not report on the outcome of the RCT [17]. Depression and anxiety [19, 20], general symptoms of psychological distress [21], impact of fibromyalgia on daily functioning [22] and health-related quality of life [23] were measured at baseline (day 1) before the measurement period as previously reported [17]. In the pre-test period, the patients were treated identically, and all experimenter interaction was with experimenters that were unaware of the patients’ group allocation. Pain intensity, pain unpleasantness, stress, and anxiety were measured daily using SMS from the mobile phones of the patients. In the morning (9 AM), afternoon (3 PM), and evening (9 PM), the patients received an SMS consisting of the following 4 questions: “what is your pain level now?,” “how unpleasant is the pain now?,” “how tense are you now?,” and “how anxious are you now?.” The response to the questions was delivered through a reply SMS containing the Numeric Rating Scale (NRS) values (0–10). If no response was obtained after 15 minutes of receiving the SMS, a reminder was sent. Responses obtained within 2 hours were considered valid.

Responses with invalid formatting were answered with an SMS containing instructions for correct formatting. To make the scoring more intuitive, the patients were supplied with a visual analogous scale with a sliding indicator in the initial consultancy with a clinical psychologist. By flipping the scale, a numeric value that corresponded to their analogous rating was read and used to translate the visual analog scale into the NRS. All of the patients who showed interest in participating had access to an SMS-compatible mobile device, which was expected because the prevalence of mobile phones in the general adult Norwegian population is close to 100%.

#### Meteorological data

All meteorological data were obtained from the Norwegian Meteorological Institute of Tromsø (MET, latitude: 69.6537, longitude: 18.9373), from 28.11.2011 to 4.8.2013. The station was operated in compliance with the ICO-certified quality control procedure Obskval. There were no recorded deviances in the meteorological parameters employed in this study. A test conducted in December 2011 concluded that the measurement precision in the station resided well within the tolerances recommended by the World Meteorological Organization (WMO). Air temperature (T) was measured at 2m above ground level using a standard PT100 sensor. Relative humidity (RH) was measured at 2 m above ground level using a HMP45D (Vaisala, Finland). Atmospheric pressure (BMP) at station ground level was measured using a digital barometer PTB220A (Vaisala). Observation values at a given time were based on 12 single measurement values in the last minute of the hour. The measure points corresponded to the time points for the SMS-pain and stress reports. A simple filtering technique to avoid noise was employed. For BMP, the observation value was produced in the instrument. For T and RH calculations were done in a Data Processing Unit (DPU). The DPU at the station was changed from a Milos 500 (Vaisala) to a CR1000 (Campbell, USA) in December 2011 due to an ordinary maintenance program, but there was no change in the calculation procedure. The participants in this study lived at a maximum of 60km from the weather station.

### Statistical analyses

All analyses were performed with SPSS v.25 (IBM, SPSS, USA). The distribution of data for pain intensity and pain unpleasantness was inspected by histograms, Q-Q plots, box-plots, and were found to be similar to normal distributions. The association between pain, emotional status and weather were tested with linear mixed models (LMM). LMM were chosen because these methods are suitable for analyzing data with unequal group sizes, handles missing data without losing power in the analyses compared to standard general linear models, and allows combinations of both fixed and random effects [24]. Pain intensity, pain unpleasantness, stress, and tension were used as dependent variables in separate analyses. Weather data, time and measures of emotional responses were entered as continuous covariates for pain outcomes, whereas weather data and time were entered as continuous covariates for stress and tension. Weather data was mean centered when used in the LMMs. Patients were assumed to induce individual variance over time, and the individual variance of each patient was treated as a random factor. Group comparison between the BMP pain-increase group and the BMP pain-decrease group was performed with independent samples t-tests. Levenes test was used to test equality of variances, and no significant differences were found. Cluster analysis to identify sub-sets in the data was performed with unsupervised Cluster node analysis using the Schwarz’s Bayesian Criterion followed by supervised K-Means cluster analysis with analysis of variance to validate the clusters found in the unsupervised cluster analysis. Moderator and mediator analyses on the effect of stress and BMP on pain reports were performed as regression analyses in LMMs. Correlation analyses were performed with Pearson correlations. An alpha-level of .05 was used for all analyses. To adjust p-values for multiple comparisons in the t-tests, the False-Discovery Rate (FDR) procedure [25] was employed by using a script for SPSS http://www-01.ibm.com/support/docview.wss?uid=swg21476447.

## Results

### Descriptive statistics

Descriptive data for pain and emotional measures are shown in Table 1. Data for weather conditions during the period of measurement is summarized in Table 2. The median number of complete SMS reports per patient containing all four measures of pain and emotional data were 30 (minimum = 18, maximum = 121; Mean = 34.12, SD = 23.33). 2712 complete SMS reports were obtained totally.

**Table 1:** Descriptive statistics and group-comparison at baseline.

	All patients (N = 48)		BMP-pain increase group (N = 8)		BMP-pain decrease group (N = 40)		Group comparison	
	Mean (SD)	Min–Max	Mean (SD)	Min–Max	Mean (SD)	Min–Max	t	p
**Gender, number**	45 female / 3 male	-	7 female / 1 male	-	38 female / 2 male	-	-	-
**Age**	48.6 (9.6)	30–73	51.25 (10.89)	37–70	47.56 (9.63)	30–73	-.93	.36
**Baseline pain intensity**	5.12 (1.53)	2.33–7.98	5.24 (1.78)	3.25–7.72	5.09 (1.5)	2.33–7.98	-.24	.81
**Baseline pain unpleasantness**	4.79 (1.66)	1.18–8.17	5.1 (2.07)	1.6–8.17	4.73 (1.59)	1.18–7.19	-.53	.60
**Baseline Stress**	1.83 (1.76)	0–7.52	.99 (1.27)	0–3.13	1.99 (1.81)	0–7.52	1.48	.15
**Baseline Tension**	.83 (1.35)	0–5.54	.91 (1.35)	0–3.53	.82 (1.37)	0–5.54	-.16	.87
**Depression—HADS**	5.75 (3.29)	0–14	4.43 (2.07)	2–8	6 (3.43)	0–14	1.16	.25
**Anxiety—HADS**	6.68 (3.69)	0–16	3 (2.52)	0–7	7.38 (3.47)	0–16	3.17	.003*
**Global Severity Index–SCL-90R**	.81 (.42)	.19–1.83	.63 (.27)	.4–1.16	.85 (.43)	.19–1.83	1.27	.21
**Fibromyalgia impact—FIQ**	52.3 (14.72)	11.49–77.47	51.39 (9.87)	32.4–60.65	52.47 (15.54)	11.49–77.47	.18	.86
**HRQL** **SF-36 Physical**	33.01 (7.12)	19.1–49.5	29.93 (7.79)	19.1–38	33.62 (6.95)	23.4–49.5	1.26	.21
**HRQL SF-36 Mental**	45.29 (12.39)	13.1–61.7	51.46 (9.03)	38.5–59.1	44.09 (12.69)	13.1–61.7	-1.46	.15

HADS = Hospital Anxiety Depression Scale. SCL90R = Symptom Checklist 90 Revised. FIQ = Fibromyalgia Impact Questionnaire. HRQL = Health Related Quality of Life, SF-36 = Short-Form 36. BMP = Barometric Pressure.

* = Significant (p < .005) after False-Discovery Rate adjustment with q < .05.

**Table 2:** Meteorological variables during the period of measurement, obtained at the Norwegian Meteorological Institute of Tromsø, latitude: 69.6537, longitude: 18.9373.

Meteorological variables	Mean (SD)	Min—Max	Median	Mode
**Barometric pressure–milibar (mbar)**	1012.65 (16.06) mbar	965–1047.7 mbar	1013.3 mbar	1031 mbar
**Air temperature–degrees Celsius (°C)**	-1.85 (5.02)°C	-18.2–27.4°C	-2.6°C	-3°C
**Relative humidity–percent (%)**	74.92 (13.99) %	22–95%	76%	92%

Period of measurement: 28.11.2011 to 4.8.2013.

### Pain and emotional data

Pain intensity and pain unpleasantness were highly correlated (Pearson r = .91, p < .001), and the LMMs showed comparable results (Table 3). Both barometric pressure and relative humidity had a significant impact on the individual slopes for pain reports, but with small parameter estimate values, B = -.003 (pain intensity) and -.007 (pain unpleasantness) for BMP, and B = .003 (pain intensity and pain unpleasantness) for humidity, see Table 3. Thus, a decrease in barometric pressure was associated with increased pain, whereas an increase in relative humidity was associated with higher pain reports. The temperature measured concomitantly with pain reports had no significant main effect on perceived pain. The interaction between BMP by humidity reached significance in the pain intensity data, but was non-significant in the pain unpleasantness data. Higher BMP and higher levels of humidity were associated with increased pain intensity, whereas humidity had no impact on pain intensity levels when BMP was lower. The interaction between BMP and temperature was significant in both the pain intensity and the pain unpleasantness data. The combination of lower BMP and reduced temperature was associated with heightened pain unpleasantness compared to when BMP was higher and increased temperature. No other interactions including the three-way interaction between the weather variables reached significance. Reported stress, but not tension was significantly associated with the slopes for both pain intensity and pain unpleasantness. Patients individual pain intensity and pain unpleasantness reports varied significantly across the measurement period shown by the random effect covariance parameters for patient ID (Pain intensity: B = 2.10, 95% CI (B) = 1.38–3.18, Wald Z = 4.73, p < .001; Pain unpleasantness: B = 2.37, 95% CI (B) = 1.56–3.58, Wald Z = 4.73, p < .001). Thus, significant individual differences were present in the data.

**Table 3:** Linear mixed model analysis for pain intensity and pain unpleasantness reports.

Parameter	B	95% CI for B Lower Upper		SE	df	t	p
***Pain intensity*:**							
**Intercept**	4.40	3.96	4.84	.22	50.32	20.19	< .001
**BMP**	-.003	-.007	-.0004	.002	2695.60	-1.75	.04
**Humidity**	.003	.001	.007	.002	2695.60	2.20	.03
**Temperature**	.0007	-.01	.01	.006	2687.43	.10	.92
**Time**	-.0003	-.002	.001	.001	2672.67	-.37	.71
**Stress**	.36	.321	.411	.02	2697.71	16.20	< .001
**Tension**	.03	-.043	.099	.02	2643.95	.75	.45
**BMP by Humidity**	.0002	.00002	.0005	.0001	2664.23	2.23	.03
**BMP by Temperature**	-.0009	-.002	-.0002	.0004	2667.42	-2.38	.02
**Humidity by Temperature**	-.0002	-.0008	.0003	.0003	2658.64	-.91	.36
**BMP by Humidity by Temperature**	.000002	.000003	.000008	.000003	2657.38	.82	.41
***Pain unpleasantness*:**							
**Intercept**	4.02	3.55	4.48	.23	50.66	17.33	< .001
**BMP**	-.007	-.01	-.003	.002	2696.51	-3.25	< .001
**Humidity**	.003	.0008	.008	.002	2660.67	2.40	.02
**Temperature**	.007	-.007	.02	.007	2673.45	.97	.33
**Time**	-.002	-.005	-.0002	.001	2676.69	-2.18	.03
**Stress**	.42	.38	.47	.02	2696.19	17.51	< .001
**Tension**	.019	-.06	.10	.04	2634.64	.50	.62
**BMP by Humidity**	.0002	-.000002	.0004	.0001	2664.23	1.81	.07
**BMP by Temperature**	-.001	-.002	-.0003	.0004	2668.03	-2.88	.004
**Humidity by Temperature**	-.00008	-.0006	.0005	.0003	2658.92	-.28	.78
**BMP by Humidity by Temperature**	.000002	-.000003	.000008	.000003	2657.62	.74	.46

BMP = Barometric Pressure. CI = Confidence Interval.

**Fig 1:**
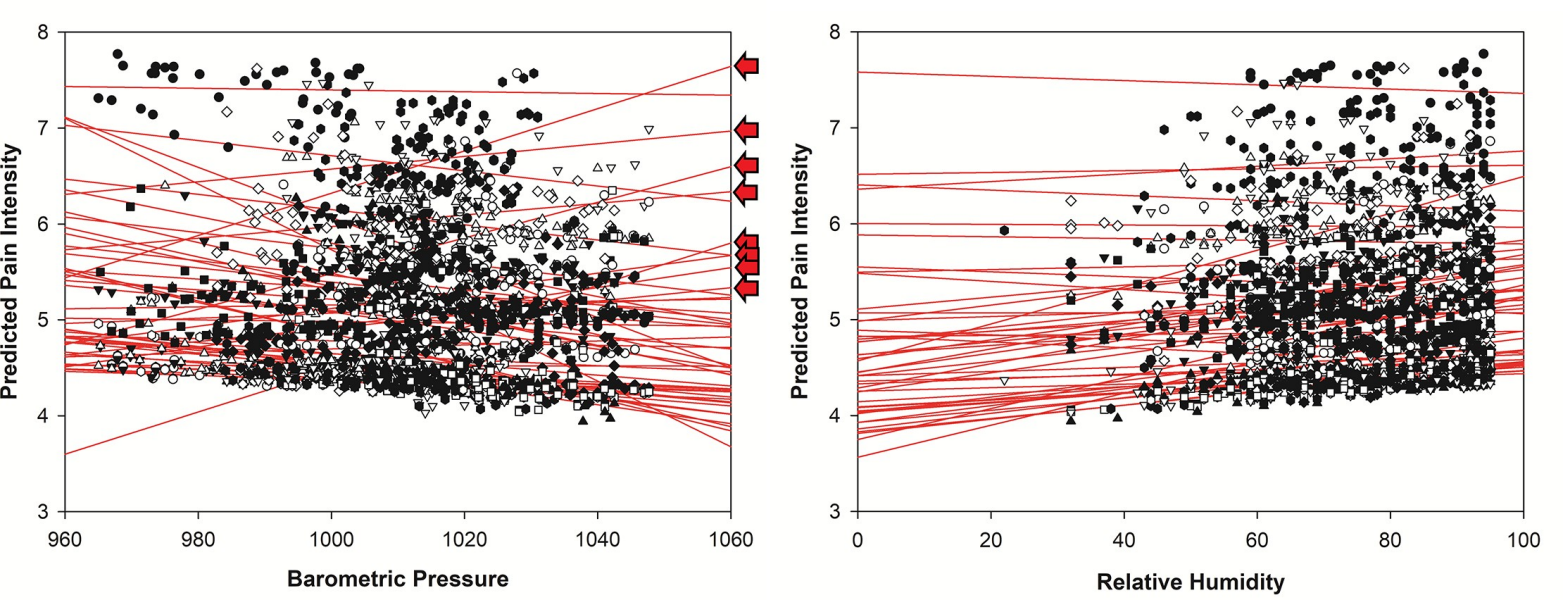
The relation between pain intensity, barometric pressure and relative humidity. Predicted pain intensity was measured on a Numeric Rating Scale from 0–10. Barometric pressure in millibar, relative humidity in percentage. Arrows depict patients (n = 8) with an opposite response in pain reports compared to the majority of the patients (n = 40). Increased barometric pressure was the only weather parameter that significantly affected emotional measures. However, similarly to the pain data, the parameter estimates were small (stress: B = .007, t = 3.97; Tension: B = .003, t = 2.7, both p-values < .005), and increased barometric pressure were associated with elevated negative emotions. The emotional measures of stress and tension varied significantly within patients over the measurement period (Stress: B = .00041, 95% CI (B) = .00026–00067, Wald Z = 4.03, p < .001; Tension B = .00007, 95% CI (B) = .00004–0001, Wald Z = 3.17, p = .002).

As evident by inspecting the individual slopes in Fig 1, eight patients reacted opposite to an increase in BMP compared to the majority (40 patients) who reported less pain with increased BP. To test the validity of dividing the sample in two clusters based on the pain and stress reports adjusted for BMP, unsupervised cluster analyses were performed. Both the pain intensity- and the pain unpleasantness data based on the LMM revealed a two-cluster structure based on the unsupervised Cluster node analysis, with a good cluster quality (Silhouette measure) of .9 for both pain intensity and pain unpleasantness. Supervised cluster analyses (K-means cluster) supported a two cluster solution for both the pain intensity data (F (1, 2710) = 25286.95, p < .001) and the pain unpleasantness data (F (1, 2710) = 25844.66, p < .001). To test possible differences between the eight patients reporting an opposite pattern of pain levels compared to the majority of the sample, baseline data obtained before the measurement period were compared. Symptoms of depression and anxiety, general psychological distress, pain intensity and unpleasantness, impact of fibromyalgia on daily functioning and health related quality of life presented in Fagerlund, Hansen & Aslaksen [17] were compared between groups. The only significant difference between the sub-group of eight patients and the majority of the sample was the lower level of anxiety in the sub-group of eight patients (t (1, 47) = 3.17, p = .003) compared to the other 40 patients.

**Fig 2:**
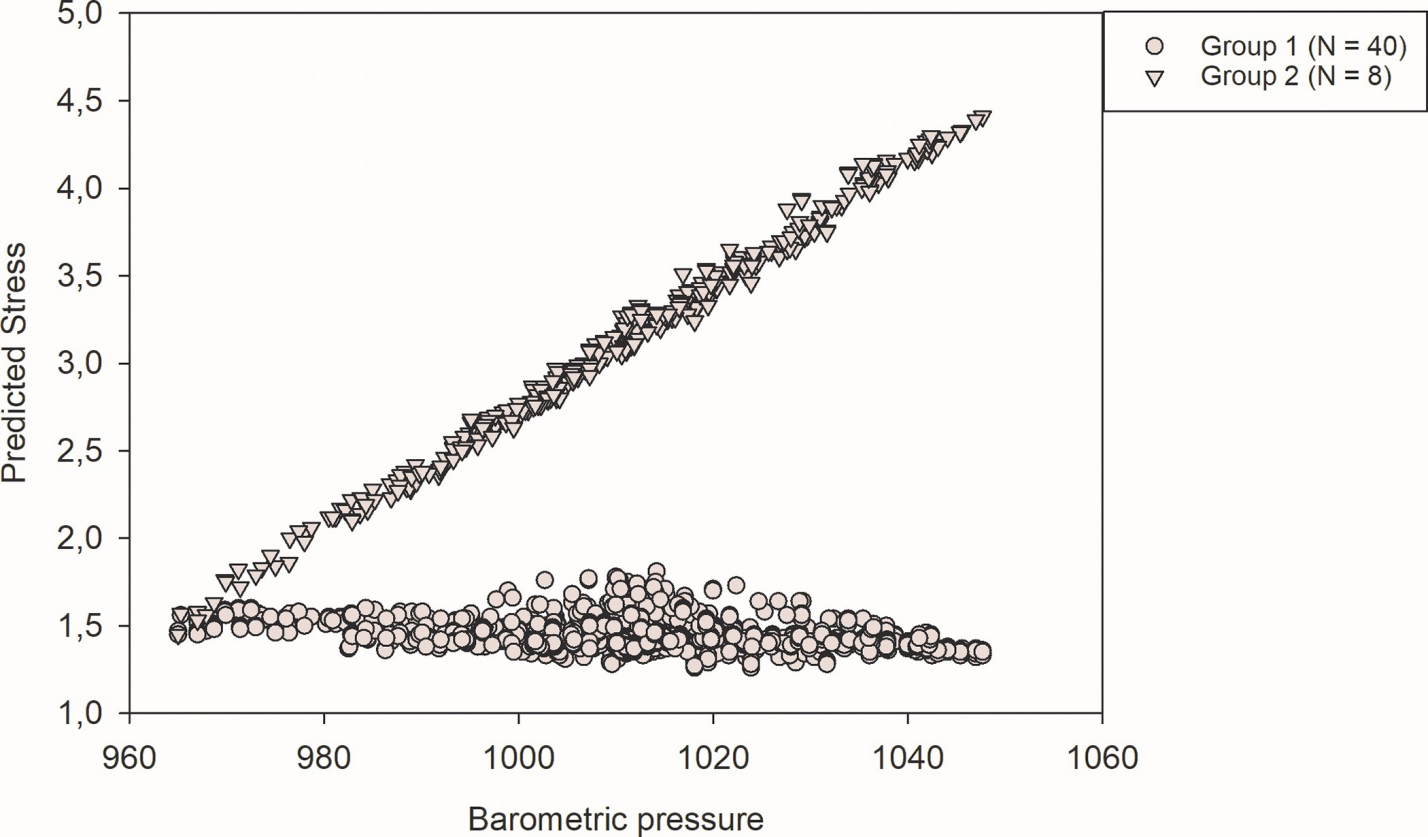
Group-difference in stress associated with barometric pressure. Barometric pressure shown in millibar. Stress was measured on a Numeric Rating Scale from 0–10. Group 1 is the majority of patients who reported lower pain concomitantly with an increase in barometric pressure, whereas group 2 is the sub-group of patients reporting increased pain concomitantly with increased barometric pressure.

To test a possible moderator effect of stress on the relationship between pain and weather data, the interaction between stress and barometric pressure together with the main effect of these variables were entered as predictors for pain intensity and pain unpleasantness in separate LMMs. Likewise, the same analyses were performed with humidity as the predictor variable and stress as the possible moderator. The only significant moderator effect of stress on pain reports was found in the pain intensity data, where stress levels moderated the impact of barometric pressure on pain intensity reports, see Table 4. Furthermore, to test the possibility that a subgroup of patients reacted opposite to change in barometric pressure with increased stress, a LMM analysis where those who reported increased pain when BMP increased (see Fig 2) was compared to those who reported lower pain when the BMP was higher. The interaction between group and BMP (B = -.04, 95% CI (B) = -.05 to -.03, SE = .004, t = -11.19, p < .001) showed that the majority of the patients reported decreased stress or no change in stress concomitantly with increased BMP, whereas the subgroup (n = 8) reported increased stress when BMP increased. Possible mediator effects of the relation between BMP, stress, pain intensity and pain unpleasantness were tested with regression analyses in the LMM. There was no evidence that stress acted as a mediator between BMP and pain intensity or pain unpleasantness, see Table 4.

**Table 4:** Moderator analysis for pain intensity and pain unpleasantness reports.

Parameter	B	95% CI for B Lower Upper		SE	df	t	p
***Pain intensity***							
**Intercept**	4.33	4.24	4.42	.03	2708	94.47	< .001
**BMP**	.003	-.002	.008	.02	2708	1.14	.25
**Stress**	.39	.35	.42	.018	2708	21.88	< .001
**BMP by Stress**	.002	.0008	.004	.001	2708	2.04	.041
***Pain unpleasantness***							
**Intercept**	9.06	4.07	14.06	2.55	2707.70	3.56	< .001
**BMP**	-.005	-.01	-.0001	.003	2700.68	-2.02	.044
**Stress**	1.90	.09	3.70	.92	2705.38	2.06	.04
**BMP by Stress**	-.001	-.003	.0003	.0009	2704.92	-1.59	.11

BMP = Barometric Pressure.

## Discussion

The results from the present study showed that data for pain levels, emotional measures and weather conditions were significantly associated. Similar to previous findings [2–5], the impact of weather was statistically significant, but the clinical impact is questionable. Although significant, the parameter estimates for both main effects and the interaction terms were small and suggest that the measured weather variables have a modest impact on self-reported chronic pain in fibromyalgia at the group-level. Furthermore, the interaction between BMP and temperature had a significant impact on both pain intensity and pain unpleasantness, where lower BMP and lower temperature were associated with higher pain reports compared to when both BMP and the temperature was higher. However, the influence of temperature on pain was reduced at higher BMP values. Nonetheless, the random effect parameter for individual variance was significant, suggesting that significant individual differences among the patients were present. Emotional data obtained concomitantly with pain reports were significantly associated with BMP, but neither humidity nor temperature had any impact on the affective measures. Similar to the analyses of pain intensity and pain unpleasantness, the parameter estimates were significant but small for BMP as a predictor. Pain was higher when pressure was low, and this effect was significantly moderated, and not mediated by stress levels. The finding that stress levels moderated the relation between BMP and pain intensity suggests that changes in BMP directly influences pain levels, but also directly heightens negative emotions. Thus, our results suggests that lower BMP increases both pain and stress independently in patients with fibromyalgia. Nonetheless, the relationship between pain and stress is reciprocate, and the present data cannot be conclusive about the causal direction of this relationship.

When examining the individual slopes for pain intensity across the scales for pressure and humidity (Fig 1), it was evident that some individuals (n = 8) reacted opposite to changes in BMP compared to the majority (n = 40). The sub-division of the sample was supported by the cluster analyses. This identification of a sub-group of patients was not part of our a-priori hypotheses, but could to some extent explain the modest impact of BMP on pain for the patient group as a whole. This sub-group of patients responded with increased stress and pain when the barometric pressure increased. The only baseline difference between those who reported increased stress and pain as the BMP increased was the level of reported anxiety before the measurement period, where the BMP pain-increase group had significantly lower anxiety levels compared to those who responded with pain decrease to increased BMP. Hence, this group showed an opposite emotional reactivity to BMP changes than the majority of the sample. In fact, the members of the sub-group reported increased stress to increased BMP whereas the majority reported little or no change when BMP increased (see Fig 2). This finding suggests that individual factors in emotional status in fibromyalgia patients are associated with responsiveness to changes in barometric pressure. However, the present data cannot inform about the causality due to the correlational design of the present study. Nonetheless, at an individual level, the possibility that meteorological conditions may affect pain and emotional levels in patients with fibromyalgia cannot be excluded. Fibromyalgia is a complex condition with several possible causes of pain [26], and the possibility exists that some individuals are physiologically affected by changes in meteorological conditions. It could be argued that the northern parts of Norway is extreme concerning weather conditions and the possible impact of weather on pain. Nonetheless, studies performed at more southern latitudes shows similar findings as the present study [2–5]

If there is a causal link between weather and pain, the pathophysiological mechanisms are still unclear. However, studies on animals have suggested that cytokine pathways involved in pain sensation may be affected by changes in hydrostatic pressure [27], and thereby also change pain perception during changes in BMP [5]. However, to our knowledge, no study has tested this hypothesis in humans. Previous findings suggests that neuroinflammatory responses may be related to cerebral glia-dysfunction in human fibromyalgia [5, 28]. Thus, the relation between individual differences in human cytokine pathways and BMP reactivity could be explored in future studies. In rats with induced neural damage, it has been shown that low barometric pressure and low temperatures increases pain behavior [11, 29], and it is suggested that the increased pain is caused, at least partly, by aggravated activity of the sympathetic nervous system. Likewise, as observed in the present study, some individuals may react with stress to changes in BMP and thereby experience heightened pain levels. Furthermore, the attributional style of the patients could influence pain reports, i.e., if someone holds the belief that bad weather causes pain increase, then the observation of weather conditions may be a self-fulfilling prophecy [30].

A novel aspect of the present study was the inclusion of measures for stress and tension that were collected simultaneously with pain reports by SMS, which may have had some advantages. Firstly, this method of obtaining longitudinal subjective reports yielded very high response rates (over 90%). Secondly, the patients found the reporting procedure to be convenient and to have minimal interference with their daily routines, giving low drop out. Finally, and important for the present study, only reports received within two hours of the time point that corresponded with the time for meteorological observations were deemed valid. Thus, the temporal similarity between subjective reports and meteorological observations was assured. Both the experimenters and patients in the present study were uninformed about the plan to investigate the effects of meteorological variables on pain and stress levels. The weather data were exported from the meteorological database after the collecting of subjective reports from the patients were complete. Thus, it was assured that the patients response patterns were not influenced by a wish to please the experimenters with regards to the hypothesis, nor under confirmation bias with regards to their subjective beliefs about the effects of weather conditions on pain or stress.

The sample size is a limitation for interpreting the results from the present study. Even if the results showed a significant contribution of individual differences based on a large number of observations, the sample consisted of only 48 patients. Future studies should also control for the attributional style of the participants with regard to the relation of weather and pain, in order to rule out the possibility of confirmation bias.

## Conclusions

In summary, the present study suggests that barometric pressure influence pain in fibromyalgia but on an individual basis that is associated with emotional factors.
